# Prevalence and correlates of overweight and obesity among school children in an urban district in Ghana

**DOI:** 10.1186/s40608-019-0234-8

**Published:** 2019-04-01

**Authors:** Theodosia Adom, Anniza De Villiers, Thandi Puoane, André Pascal Kengne

**Affiliations:** 1 Nutrition Research Centre, Radiological and Medical Sciences Research Institute, Ghana Atomic Energy Commission, Accra, Ghana; 2 School of Public Health, Faculty of Community and Health Sciences, University of the Western Cape, Cape Town, South Africa; 3 Non-communicable Disease Research Unit, South African Medical Research Council, Cape Town, South Africa

**Keywords:** Ghana, Obesity, Overweight, School children

## Abstract

**Background:**

There is limited data on risk factors associated with childhood overweight and obesity in Ghanaian school children. Therefore, the aim of this study was to assess the prevalence of overweight and obesity and associated risk factors in Ghanaian school children.

**Methods:**

Data for this study were obtained from a cross-sectional survey of 543 children aged 8 and 11 years, attending private and public primary schools in the Adentan Municipality of Greater Accra Region, Ghana. Anthropometric, dietary, physical activity, sedentary behaviours, sleep duration and socio-demographic data were collected. BMI-for-age Z-scores were used to classify children as overweight/obesity. Multivariable logistic regressions were used to assess the determinants of overweight and obesity.

**Results:**

The overall prevalence of overweight/obesity was 16.4%. Children living in middle (OR = 1.88; 95% CI = 1.01–3.50) and high socioeconomic status (SES) households (2.58; 1.41–4.70) had increased odds of being overweight or obese compared to those living in low SES household. Attending private school (2.44; 1.39–4.29) and watching television for more than 2 h each day (1.72; 1.05–2.82) were significantly associated with increased likelihood of overweight and obesity. Children who slept for more than 9 h a night (0.53; 0.31–0.88) and walked or cycled to school (0.51; 0.31–0.82) had lower odds of being overweight or obese.

**Conclusions:**

A number of modifiable risk factors were associated with overweight and obesity in this study. Public health strategies to prevent childhood obesity should target reduction in television watching time, promoting active transport to and from school, and increasing sleep duration.

## Background

The increasing prevalence of childhood obesity continues to be a major public health concern globally [1–3]. In 2013, the Global Burden of Disease estimated that 23.8% of boys and 22.6% of girls in developed countries were overweight or obese [3]. During the same period, the prevalence increased from 8.1 to 12.9% in boys and from 8.4 to 13.4% in girls in developing countries [3]. In sub-Saharan Africa where countries are still developing, there is growing evidence of increasing overweight and obesity among school-aged children [4, 5].

The health implications of overweight and obesity cannot be ignored [6]. Children with obesity are at increased risk of developing hypertension, high cholesterol, orthopaedic problems, and type 2 diabetes in childhood [6–8], which may persist in adulthood [9]. There is also considerable evidence that overweight and obesity in childhood predict adult obesity [10]. Preventing unhealthy weight gain from an early age is a public health strategy and identifying modifiable risk factors in the local context is important for a successful intervention.

Childhood obesity reflects complex interactions between the individual, behavioural and environmental factors [11]. Traditionally, long-term energy imbalance resulting from high energy intake and low energy expenditure has been implicated in the epidemiology of overweight and obesity [12]. Lifestyle and behavioural changes including consumption of diets high in refined sugars and saturated fats but low in fruits and vegetables, physical inactivity, increased sedentary behaviours [13] and short sleep duration have contributed to the increasing prevalence of overweight and obesity in children and adults [14–19]. Moreover, studies in developed and developing countries have linked socioeconomic status to obesity prevalence [4, 20, 21]. In Africa, the type of school a child attends is invariably linked to the SES of the family; children from higher SES households are more likely to attend private or affluent schools and have higher risk of becoming overweight or obese [4, 22] than children from low SES households.

In developing countries including Ghana, nutrition transition and lifestyle changes associated with rapid urbanisation, globalisation and industrialisation are major contributing factors towards unhealthy lifestyles [12, 18, 19]. There is growing evidence of increasing prevalence of overweight and obesity among children under the age of five [23] and school-aged children [24, 25] in Ghana. Findings from the Ghana Demographic and Health Survey revealed that the prevalence of obesity increased from less than 1% in 1988 to 5% in 2008 in children under the age of five [23]. During the primary school period, unhealthy weight gains may arise from energy imbalance resulting from poor dietary habits [26] and physical inactivity [24]. Despite these, older children, unlike children under 5 and adolescents are usually not targets of representative health surveys in developing countries including Ghana [23, 27]. Nationally representative data on the prevalence of overweight and obesity among school-aged children are lacking. While a number of individual studies involving Ghanaian children have reported increasing prevalence [24, 25, 28, 29], fewer studies have investigated the independent associations of determinants or associated risk factors with overweight and obesity. Therefore, the objective of this study was to determine the prevalence of overweight and obesity and to examine the associated risk factors in Ghanaian school children.

## Methods

### Study design and population

This was a cross-sectional study that involved children aged 8–11 years attending private and public primary schools in the Adentan Municipality of Greater Accra Region, Ghana. Adentan is one of the 213 districts in Ghana. There are 13 public basic schools and 135 private basic schools in the Municipality which are either exclusively primary (grades 1–6), or Primary and Junior High (Grades 1-JHS 3). The Adentan Municipality was chosen because it is one of the fastest growing municipalities and a high proportion (62.5%) of the population resides in urban areas [30].

The study was approved by the Ethical Review Committees of the Ghana Health Service and the Senate Research Committee of the University of the Western Cape. Approval was also obtained from the Municipal Education Directorate of the Ghana Education Service as well as from the Heads of all participating schools. Written informed consent were given by parents/legal guardians of children. Verbal assent was given by each participating child.

### Sampling

A minimum sample size of 206 was estimated based on an overweight/obesity prevalence of 15% among school children in urban Ghana [31], using the formula for prevalence studies [32]. This was multiplied by design effect for cluster sampling of 2 to give a sample of 412. Assuming a non-response rate of 10%, the sample size was adjusted to 453 children. Fourteen schools were selected from a list of basic schools in the district obtained from the Municipal Education Directorate of the Ghana Education Service using a random cluster sampling technique. In each school, all apparently healthy children between the ages of 8 and 11 years were eligible. Exclusion criteria were known medical conditions such as diabetes because some medications like insulin treatment in such individuals predisposes them to excess weight gain [33]. None of the children was however excluded from the study.

Simple random sampling technique (balloting) was used to select pupils from each selected school. The children were asked to pick from a bowl, papers that were pre-coded as “yes” or “no”. The number of children selected from each school ranged from 40 to 100. In each class, sampling was stratified on gender so that boys were sampled separately from girls. A total of 790 invitations and consent forms were distributed to those who picked “yes” for parental approval. Out of this, 562 returned with informed parental consent (71.1%) and were sampled.

### Data collection

#### Dietary, physical activity, sedentary behaviours and sleep duration

A pre-tested questionnaire was used to obtain data from the children by trained research assistants. Each child was given a printed form and pencil to write their responses. Questions and response options were read out in the classroom setting and the children were encouraged to answer independently under the supervision of the research assistants, who also ensured that children responded to the previous question before proceeding to the subsequent questions. This approach was adopted because of the younger children (8–9 years), as it helped them to concentrate for a longer period during data collection. Dietary data collected included eating breakfast before school, fruits and vegetables intake, fried foods, consumption of sweetened beverages and soft drinks, and eating sweets, chocolates and chips for snacks. Responses were “No”, “sometimes, 2-3 times a week”; and “Yes, more than 3 times a week” for breakfast, fried foods, of sweetened beverages and soft drinks. For snacks, fruits and vegetables, responses were “No/rarely”, and “Yes, more than 3 times a week”.

Questions on physical activity level was collected using a modified version of the International Physical Activity Questionnaire for Children [34]. Questions were grouped in 9 categories. The frequency of performing certain activities one week prior to the study was reported. Each of the 9 items was scored using a scale of 1 to 5 and the mean of the items used to calculate the final summary score. The physical activity level was categorised as: light (score < 2), moderate (score 2–4), and vigorous (score = 5) with a higher score indicating a higher level of activity [34]. The cut-off points for meeting physical activity recommendations were defined as ≥2.9 for boys and ≥ 2.7 for girls [35].

To assess the mode of transportation to and from school, children responded to “how they usually travelled to and from school in the last seven days” with response options “walking/cycling”, “bus, car, vehicle” or “both”. The responses were dichotomised as “walking/cycling” and “motorised”. Children responded to the frequency of playing video/computer games. The time spent in watching television daily was reported in hours and dichotomised as moderate (< 2 h) and excessive (≥ 2 h). Screen time was used as a measure of sedentary behaviour. Sleep duration was assessed by asking the usual time of going to bed at night and usual time of waking up in the morning on a typical weekday and weekend or holiday. We dichotomised sleep duration as short (< 9 h) and sufficient (> 9 h) based on the recommendations by the National Sleep Foundation [36].

### Socio-demographic data

A structured questionnaire was used to collect the following data on demographic and household characteristics: gender, age, parents’ occupation, household size, source of water and sanitation, and fuel for cooking. Additional data on possession of household assets including television, radio, car/motorbike, refrigerator, washing machine, telephone, computer, cable TV and microwave oven were collected.

### Anthropometry

Body weight was measured to the nearest 0.1 kg with an electron digital scale (Seca 869, GmbH & Co, Germany). Children were weighed in their school uniforms but asked to remove shoes, socks, watches, sweaters, jackets and items in the pockets. Height was measured to the nearest 0.1 cm using the Shorr Board (Shorr Productions, Olney, MD). All measurements were done in duplicates by the same research assistants. From the averages of the duplicate measures, BMI was computed as body weight (in kilogramme) divided by height (in metre square). BMI-for-age z-scores (BAZ) were used to categorise pupils as thin, normal weight, overweight or obese [37]. Data collection was done at the schools’ premises.

### Statistical analysis

Variables on source of water and sanitation, and household assets were subjected to principal component analysis. The first component was extracted to create wealth scores of the household which were then split into thirds and reported as low SES (lowest third), middle SES (middle third) and high SES (highest third) households [38]. Categorical variables were reported as counts and percentages, and mean and standard deviations for continuous variables. For group comparisons, chi-square test and student t-test were performed.

For the purposes of analysis, we collapsed overweight (pre-obesity) and obesity into one variable “overweight or obesity” and other weight categories as “non-overweight/obesity”. The proportions of children who were overweight or obese was computed as BAZ > + 1.0 SD. Multivariable logistic regressions were used to identify sociodemographic characteristic, dietary, physical activity and sedentary behaviours associated with overweight or obesity while controlling for age and gender. Variables that correlated (*p* < 0.05) or tended to be associated with the outcome variable (*p*-values < 0.1) at the univariable levels were selected and included in the models. In the multivariable analyses, only variables that were associated with overweight/obesity (p < 0.05) were retained. Covariates such as age, gender, SES, physical activity and dietary habits were forced in all multivariable models. All analyses were performed with Stata 13.0 using cases with complete data. Statistical significance was set at *p* < 0.05.

## Results

### Background characteristics

This analysis involves data from 543 children. The sociodemographic characteristics of children are presented in Table 1. The mean age of children who participated in the study was 9.8 ± 1.0 years with majority being females (62.4%). Compared with public schools, more children attending private schools live in households in the high socioeconomic category (16.2% vs 50.4%; *p* < 0.0001).

**Table 1:** Background characteristics of participating children by gender and type of school^a, b^

	Overall	Gender		*p*-value	School type		*p*-value
		Female	Male		Private	Public	
Number (%)	543 (100)	339 (62.4)	204 (37.6)	0.265	272 (50.1)	271 (49.9)	
Age (years)	9.8 ± 1.0	9.8 ± 1.0	9.9 ± 1.0		9.8 ± 1.0	9.9 ± 1.0	0.725
Age groups							
8–9	199 (36.7)	129 (38.0)	70 (34.3)	0.217	98 (36.0)	101 (37.3)	0.764
10–11	344 (63.4)	210 (62.0	134 (65.7)		174 (64.0)	170 (62.7)	
Household size				0.020			0.396
≤ 3	31 (5.7)	17 (5.0)	14 (6.9)		16 (5.9)	15 (5.5)	
4 - 6	323 (59.5)	210 (62.0)	113 (55.4)		170 (62.5)	153 (56.5)	
7 - 10	168 (30.9)	94 (27.7)	74 (36.3)		78 (28.7)	90 (33.2)	
≥ 10	21 (3.9)	18 (5.3)	3 (1.4)		8 (2.9)	13 (4.8)	
Father’s occupation				0.913		`	< 0.0001
Artisan	149 (27.4)	96 (28.3)	53 (26.0)		34 (12.5)	115 (42.2)	
Trading	58 (10.7)	36 (10.6)	22 (10.8)		42 (15.4)	16 (5.9)	
Professional^c^	181 (33.3)	112 (33.0)	69 (33.8)		138 (50.7)	43 (15.9)	
Other	117 (21.6)	69 (20.3)	48 (23.5)		42 (15.4)	75 (27.7)	
I do not know	18 (3.3)	12 (3.5)	6 (2.9)		12 (4.4)	6 (2.2)	
Unemployed/retired	20 (3.7)	14 (4.1)	6 (2.9)		4 (1.5)	16 (5.9)	
Mother’s occupation				0.311			0.003
Artisan	75 (13.8)	46 (13.6)	29 (14.2)		38 (14.0)	37 (13.7)	
Trading	290 (53.4)	187 (55.2)	103 (50.5)		142 (52.2)	148 (54.6)	
Professional^c^	99 (18.2)	57 (16.8)	42 (20.6)		65 (23.9)	34 (12.6)	
Other	35 (6.5)	23 (6.8)	12 (5.9)		12 (4.4)	23 (8.5)	
I do not know	5 (0.9)	5 (1.5)	–		2 (0.7)	3 (1.1)	
Unemployed/retired	39 (7.2)	21 (6.2)	18 (8.8)		13 (4.8)	26 (9.6)	
Socioeconomic status of household				0.639			< 0.0001
Low	181 (33.3)	116 (34.2)	65 (31.9)		41 (15.1)	140 (51.7)	
Middle	181 (33.3)	115 (33.9)	66 (32.3)		94 (34.6)	87 (32.1)	
High	181 (33.3)	108 (31.9)	73 (35.8)		137 (50.4)	44 (16.2)	

^a^Values presented as numbers and percentages; ^b^Mean (SD); ^c^Professionals include doctors, lawyers, engineers, pharmacists and teachers

*p* < 0.05

The behavioural characteristics of the children are summarised in Table 2. Fruits and vegetables intake was high (91.5 and 87.5%). Generally, the intakes did not differ by gender or school type. Compared with children who attended public schools, a significantly higher proportion of children in private schools consumed breakfast (78.3% vs 65.7%; *p* = 0.001), chips, sweets and chocolates snacks (68.7% vs 57.9%; *p* = 0.009), fried foods (56.2% vs 40.6%; *p* < 0.0001) and, sweetened beverages and soft drinks (84.2% vs 74.2%; *p* = 0.004).

**Table 2:** Behavioural characteristics of participating children by sex and type of school ^a,b^

	Overall	Sex		*p*-value	School type		*p*-value
		Female	Male		Private	Public	
Breakfast				0.343			0.001
No	152 (28.0)	95 (28.0)	57 (27.9)		59 (21.7)	93 (34.3)	
2–3 days/week	93 (17.1)	64 (18.9)	29 (14.2)		41 (15.1)	52 (19.2)	
3 or more times/week	298 (54.9)	180 (53.1)	118 (57.8)		172 (63.2)	126 (46.5)	
Fruits				0.069			0.213
No/rarely	46 (8.5)	23 (6.8)	23 (11.3)		19 (7.0)	27 (10.0)	
3 or more times/week	497 (91.5)	316 (93.2)	181 (88.7)		253 (93.0)	244 (90.0)	
Vegetables				0.084			0.808
No/rarely	68 (12.5)	36 (10.6)	32 (15.7)		35 (12.9)	33 (12.2)	
3 or more times/week	475 (87.5)	303 (89.4)	172 (84.3)		237 (87.1)	238 (87.8)	
Chips, sweets, chocolates				0.107			0.009
No/rarely	199 (36.7)	133 (39.2)	66 (32.4)		85 (31.3)	114 (42.1)	
3 or more times/week	344 (63.3)	206 (60.8)	138 (67.6)		187 (68.7)	157 (57.9)	
Fried foods				0.357			< 0.0001
No	156 (28.7)	109 (32.1)	47 (23.0)		89 (32.7)	67 (24.7)	
2–3 times/week	124 (22.8)	71 (20.9)	53 (26.0)		30 (11.0)	94 (34.7)	
More than 3 times/week	263 (48.4)	159 (46.9)	104 (51.0)		153 (56.3)	110 (40.6)	
Sweetened beverages and soft drinks				0.787			< 0.0001
No	104 (19.1)	68 (20.1)	36 (17.7)		46 (16.9)	58 (21.4)	
2–3 times/week	149 (27.4)	92 (27.1)	57 (27.9)		55 (20.2)	94 (34.7)	
More than 3 times/week	290 (53.4)	179 (52.8)	111 (54.4)		171 (62.9)	149 (43.9)	
Mean physical activity score ^b^	2.56 ± 0.56	2.55 ± 0.54	2.57 ± 0.61	0.652	2.52 ± 0.57	2.60 ± 0.57	0.116
Physical activity level				0.388			0.995
Low	91 (16.8)	52 (15.3)	39 (19.1)		46 (16.9)	45 (16.6)	
Moderate	444 (81.8)	283 (83.5)	161 (78.9)		222 (81.6)	222 (81.9)	
High	8 (1.4)	4 (1.2)	4 (2.0)		4 (1.5)	4 (1.5)	
Meeting PA guidelines				0.065			0.006
No	357 (65.8)	213 (62.8)	144 (70.6)		194 (71.3)	163 (60.1)	
Yes	186 (34.2)	126 (37.2)	60 (29.4)		78 (28.7)	108 (39.9)	
Transport to and from school				0.362			< 0.0001
Walking/cycling	330 (60.8)	201 (59.3)	129 (63.2)		138 (50.7)	192 (70.9)	
Motorised	213 (39.2)	138 (40.7)	75 (36.8)		134 (49.3)	79 (29.1)	
TV Watch duration				0.177			0.056
2 h or less/day	337 (62.1)	203 (59.9)	134 (65.7)		158 (58.1)	179 (66.1)	
More than 2 h/day	206 (37.9)	136 (40.1)	70 (34.3)		114 (41.9)	92 (33.9)	
Playing video/ computer games				0.030			0.449
No/rarely	330 (60.8)	218 (64.3)	112 (54.9)		161 (59.2)	169 (62.4)	
3 times or more/week	213 (39.2)	121 (35.7)	92 (45.1)		111 (40.8)	102 (37.6)	
Sleep duration				0.810			0.074
Less than 9 h	130 (23.9)	80 (23.60)	50 (24.5)		74 (27.2)	56 (20.7)	
9 h or more	413 (76.1)	259 (76.4)	154 (75.5)		198 (72.8)	215 (79.3)	

^a^Values presented as number and percentages; ^b^mean (SD)

*p* < 0.05

The overall mean physical activity score was 2.56 ± 0.56. The majority of the children (81.8%) engaged in moderate physical activity with only a few (1.4%) participating in high physical activity. Overall, about one-third met physical activity recommendations levels with a higher proportion of girls than boys (37.2 and 29.4%), and more children in public compared with those in private schools (39.9% vs 28.7%; *p* = 0.006). Additionally, the mode of transport to and from school differed by school type, but not by gender. A significantly higher proportion of children attending private schools used cars, vehicles or bus compared with their counterparts in public schools (49.3% vs 29.1%, *p* < 0.0001). Majority (62.1%) spent less than 2 h a day watching television and this did not differ by gender or type of school. Moreover, a higher proportion of boys than girls (45.1% vs 35.7%; *p* = 0.03) played video and computer games. The majority of the children had sufficient sleep at night; only 23.9% slept for less than 9 h a night.

### Prevalence of overweight and obesity

With regards to BMI status, 6.1% of the children were thin, 77.5% were normal weight, 9.2% overweight and 7.2% obese (Table 3). More girls (10.6%) than boys (6.9%) were overweight whereas more boys (9.3%) than girls (5.9%) were obese. Nonetheless, the observed difference was not significant. A significantly higher proportion of children attending private schools were overweight and obese (12.9 and 11.0%) compared with those in public schools (5.5 and 3.3%). Overall prevalence of overweight (and obesity) was 16.4%; 16.5% of girls and 16.2% of boys and by school type 23.9% private and 8.8% public.

**Table 3:** Anthropometric characteristics of school children by gender and type of school

	Overall	Gender		*p*-value	School type		*p*-value
		Female (*n* = 339)	Male (*n* = 204)		Private (*n* = 272)	Public (*n* = 271)	
Body mass index categories (%)				0.230			< 0.0001
Thinness	33 (6.1)	22 (6.5)	11 (5.4)		18 (6.6)	15 (5.5)	
Normal weight	421 (77.5)	261 (77.0)	160 (78.4)		189 (69.5)	232 (85.6)	
Overweight	50 (9.2)	36 (10.6)	14 (6.9)		35 (12.9)	15 (5.5)	
Obesity	39 (7.2)	20 (5.9)	19 (9.3)		30 (11.0)	9 (3.3)	

Thinness: BAZ < −2SD; Overweight: +1SD < BAZ < +2SD; Obese: + ≥2SD BAZ; (WHO, 2007)

*p* < 0.05

### Factors associated with overweight and obesity

Table 4 shows the factors associated with overweight or obesity. In the gender and age-adjusted analyses, children living in middle and high SES households were more likely to be overweight or obese compared with those living in low SES households (OR = 1.87, 1.0–3.48 and OR = 2.57, 1.41–4.68 respectively). Children who attending private schools had higher odds of overweight or obesity than those attending public schools (OR = 3.23, 1.95–5.35). It was realised that children who watched television for more than 2 h a day (OR = 2.08, 1.31–3.28) were twice likely to be overweight or obesity.

**Table 4:** Factors associated with overweight or obesity

	Odds ratio	95% CI	*p*-value	Adjusted Odds Ratio^a^	95% CI	*p*-value
*Age group*						
8–9	1	0.46–1.16				
10–11	0.73		0.183			
*Sex*						
Female	1	0.61–1.56				
Male	0.97		0.917			
*School type*						
Public	1	1.95–5.35		1		
Private	3.23		< 0.0001	2.44	1.39–4.29	0.002
*Household socioeconomic status*						
Low	1			1		
Medium	1.87	1.01–3.48	0.048	1.30	0.67–2.55	0.437
High	2.57	1.41–4.68	0.002	1.27	0.66–2.66	0.494
*Eat breakfast*						
No	1	0.51–2.00	0.984			
2–3 days/week	1.01	0.54–1.53	0.717			
More than 3 days	0.91					
*Eat fruits*						
No	1					
Yes	0.789	0.37–1.70	0.544			
*Eat vegetables*						
No	1					
Yes	0.723	0.38–1.37	0.319			
*Eat chips, sweets, chocolate snacks*						
No	1					
Yes	1.17	0.72–1.88	0.529			
*Fried foods*						
No	1	0.46–1.72				
2–3 times/week	0.89	0.65–1.89	0.728			
More than 3 times/week	1.11		0.699			
*Sweetened beverages and soft drinks*						
No	1					
2–3 times/week	1.62	0.79–3.30	0.184			
More than 3 times	1.39	0.72–2.68	0.329			
*Physical activity level*						
Low	1	0.46–1.49				
Moderate	0.83	0.07–5.40	0.529			
High	0.62		0.667			
*Mode of transport to/from school*						
Motorised	1			1		
Walking/cycling	0.39	0.25–0.62	< 0.0001	0.51	0.31–0.82	0.006
*TV watch duration*						
2 h or less/day	1	1.31–3.28		1		
More than 2 h/day	2.08		0.002	1.72	1.05–2.82	0.031
*Sleep duration*						
8 h or less	1	0.25–0.66		1		
9 h or more	0.41		< 0.0001	0.53	0.31–0.88	0.015

^a^Models adjusted for: age, sex, SES, physical activity and dietary habits

*p* < 0.05

Active transport to and from school (OR = 0.39, 0.25–0.62) was associated with decreased likelihood of overweight or obesity as compared with motorised transport. Additionally, longer sleep duration (for at least 9 h a night) was similarly associated with decreased odds of overweight or obesity (OR = 0.41, 0.25–0.66).

Intake of sweetened beverages and soft drinks, chips, sweets and chocolate for snack, breakfast, fruits and vegetables, and overall physical activity were not significantly associated with overweight or obesity.

After adjusting for SES, age, gender, physical activity and dietary habits, attending private school (AOR = 2.44, 1.39–4.29) and watching television for at least 2 h a day (AOR = 1.72, 1.05–2.82) were significantly associated with increased likelihood of overweight and obesity whereas sleeping for at least 9 h a night (AOR = 0.53, 0.31–0.88), and using active transport to and from school were associated with decreased odds of overweight and obesity.

## Discussion

The results of this study highlights the burden of overweight and obesity, the behavioural and sociodemographic correlates in school children aged 8–11 years living in an urban area in Ghana. Among these urban children, the overall prevalence of overweight or obesity was 16.4%, supporting results from previous studies in South Africa, Tanzania and Kenya [5, 39–41], and also in developed countries [20, 42]. In a study involving Kenyan urban school children aged 9–11 years, an overweight/obesity prevalence of 20.8% was reported [41]. In another study in South Africa, overweight or obesity prevalence of 22.9% was reported among school children aged 7–18 years [5]. While the prevalence in the present study may be lower than what had been reported in other countries, the results has demonstrated the burden of overweight and obesity in Ghanaian school children considering that the participants in the present study were younger (8–11 years). The observed trend is mostly consistent with the nutrition transition and urbanisation in the region [21].

Positive associations were found between overweight or obesity, SES and attending private school, a finding supportive of results from previous studies [22, 24, 41, 43]. Results from a recent systematic review of 20 studies involving school-aged children from sub-Saharan Africa suggested that children from the highest SES households and attending affluent or private schools were significantly more likely to be overweight or obese [22].

High SES households in developing countries may have access to high energy foods and drinks and processed foods compared with low SES households [44, 45]. Moreover, there may be increased use of technology [45] such as cars, electronic devices and indoor entertainments facilities like gaming consoles and televisions in the high SES households compared to low SES households. In this study, significantly higher proportion of children attending private schools were from higher SES households, consumed high energy foods, snacks and drinks, and used motorised transport to and from school compared to children attending public schools, consistent with the aforementioned findings. Additionally, more children in private schools were engaged in sedentary activities compared to those in public schools, though not significant. These dietary and sedentary habits could contribute to weight gain among the children [39]. The results from this study however contrast observations from developed countries where inverse associations exist between SES and overweight or obesity prevalence [42, 46, 47]. This has been attributed to low consumption of healthy foods [48], less physical activity and high sedentary behaviours in low SES groups [49].

The present study did not observe an association of overweight or obesity with overall physical activity participation. However a significant inverse association was found with active transport to and from schools, consistent with previous studies [24, 39, 50, 51]. For example in the International Study of Childhood Obesity, Lifestyle and Environment (ISCOLE) multinational study involving 12 countries, active transport was associated with lower risk of obesity [50]. Children who engage in active transport to school had higher odds of meeting moderate-to-vigorous physical activity recommendations [41, 52, 53]. However other studies also reported conflicting results of an association [13].

There is epidemiological evidence linking sleep duration to overweight or obesity in children and adolescents [14–16, 54]. In the present study, a significant inverse association was found between sleep duration and overweight or obesity, an observation consistent with previous studies [16, 55, 56]. For example results from one recent systematic review and meta-analysis indicate short sleep duration increases the risk of childhood obesity [14]. Likewise, results from cross-sectional and prospective studies highlighted consistent inverse associations between sleep duration and the risk of obesity [54]. The underlying mechanisms through which sleep influences weight status are not well-understood. Results from 29 studies conducted in 16 countries involving children and adolescents suggest changes in food intake pathways [54] and excessive media use [57] may play a role. Shorter sleep duration may result in several hormonal changes and metabolic abnormalities [54]. While available evidence suggests that sleep influences weight status through increased appetite, hunger and food intake [58, 59], the evidence in support of reduced energy expenditure through decreased physical activity and increased sedentary behaviour is conflicting [60].

The association of television viewing time and overweight or obesity observed in this study is consistent with previous studies [16, 61–64]. Results from a large sample of children and adolescents from mostly low-to-middle income countries demonstrated a positive association between television time and BMI [61]. Similarly, results from a recent meta-analysis of 14 cross-sectional studies and 24 reports involving 106,169 children suggested that increased TV watching is associated with higher risk of childhood obesity [63]. Also, findings from the review by Tremblay et al. [64] indicated that watching television for more than two hours a day was associated with unfavourable health outcomes including body composition, of children and youth. Although it does not capture the whole spectrum of sedentary behaviour, television viewing which is most commonly used as a proxy for sedentary behaviour in children and adolescents [64], appears to be more closely associated with weight status in children.

Several potential mechanisms have been proposed to explain the link between television viewing and obesity. These include limited time available to engage in outdoor games leading to overall reduced physical activity [65, 66], increased energy intake via snacking while watching television as well as exposure to advertisements involving high energy foods and snacks [66, 67]. Among US children, hours of television watch was positively associated with total energy intake and inversely associated with physical activity [66]. Evidence from Australia, the USA and eight European countries suggests an association between overweight in children and the number of advertisements per hour on children television, particularly those advertisements that promote the consumption of energy-dense, nutrient-poor foods [67]. It is however worth noting that playing video and computer games was not associated with being overweight or obese in the present study contrary to results from other studies.

Unlike other studies [19, 47, 68–70], no evidence of an association was found among overweight/obesity with dietary intake and overall physical activity. Physical education and physical activity are mandatory requirement of the basic school curriculum in Ghana and as such all healthy school-going children are expected to fully participate. Nonetheless, data available suggest that 50% of Ghanaian children and adolescents in schools engage in sports [71]. In the present study, the proportion of children who met recommended physical activity guidelines was 34.2% consistent with results from Physical Activity Report Card [71], suggesting low physical activity participation. Despite not meeting the guidelines, it is possible majority of the children engaged in games and sporting activities during and after school hours. This could partly explain the lack of association between physical activity and overweight or obesity.

### Strengths and limitations

The present study was conducted in an urban district hence the findings can be generalised to apparently healthy children in urban schools in Ghana. Another strength of the study was the objective measurement of weight and height as opposed to self-reported anthropometric data. Further, physical activity was assessed using PAQ-C, a validated tool for physical activity assessment among children in the school settings. Additionally, the sleep duration cut-off points used were based upon guidelines of a well-recognised organisation.

There were limitations to the interpretation of the findings of the current study. Firstly, the study design was cross-sectional and inferences of causality could not be made. Secondly, self-reported data was obtained from children and this has its inherent challenges including: social desirability, recall bias and misreporting [72]. The lack of association with dietary intake could be attributed to inaccuracies in measuring dietary data by recall, and under-reporting of snack, fatty foods, and foods rich in carbohydrates, commonly observed in obese individuals [72]. Since data was not collected on quantities of foods and drinks consumed, it is possible that children in the overweight/obesity category consumed these foods less often but in higher quantities. In addition, grouping the wealth score of the households into thirds may have resulted in ranking the poor and the very poor incorrectly. Nevertheless this methodology is reliable in ranking household SES status in these settings in the absence of income and expenditure data [38]. Another potential limitation worth noting was that the multilevel structure of the data was not accounted for in the analyses. Although several potential covariates including physical activity and dietary habits were controlled for in the analyses, these were not objectively assessed. Data on an important confounder, parental BMI was not collected. Despite these limitations, the data collected was reliable and reflected what had been reported in the literature on the issues of childhood overweight and obesity.

## Conclusion

Changes in economic status and sociodemographic profiles associated with urbanisation favour lifestyle behaviours shifts towards less physical activity, increased sedentary habits and unhealthy dietary patterns. Several correlates such as attending private school, short sleep duration, high level of television watching, and motorised transport were associated with overweight and obesity among school children in urban Ghana. Public health interventions to address childhood overweight and obesity could target both homes and schools. Watching television may represent one important area of intervention targeting obesity prevention in children. At the home settings, parents could consider restricting time spent in watching television among school children. We recommend the promotion and support of regular active transport by the schools and families.

## Abbreviations

AOR: Adjusted odds ratio
BAZ: Body mass index for age Z scores
BMI: Body mass index
CI: Confidence intervals
ISCOLE: International Study of Childhood Obesity, Lifestyle and Environment
OR: Odds ratio
PA: Physical activity
PAQ-C: Physical activity questionnaire for children
SD: Standard deviation
SES: Socio-economic status
WHO: World Health Organisation

## References

[1] de Onis M, Blössner M, Borghi E (2010). Global prevalence and trends of overweight and obesity among preschool children. Am J Clin Nutr.

[2] Lobstein T, Baur L a, Uauy R (2004). Obesity in children and young people: A crisis in public health. Obes Rev.

[3] Ng M, Fleming T, Robinson M, Thomson B, Graetz N, Margono C (2014). Global, regional, and national prevalence of overweight and obesity in children and adults during 1980–2013: a systematic analysis for the Global Burden of Disease Study 2013. Lancet.

[4] Muthuri SK, Francis CE, Wachira L-JM, LeBlanc AG, Sampson M, Onywera VO (2014). Evidence of an overweight/obesity transition among school-aged children and youth in sub-Saharan Africa: a systematic review. PLoS One.

[5] Negash S, Agyemang C, Matsha TE, Peer N, Erasmus RT, Kengne AP (2017). Differential prevalence and associations of overweight and obesity by gender and population group among school learners in South Africa: a cross-sectional study. BMC Obes.

[6] Reilly JJ (2003). Health consequences of obesity. Arch Dis Child.

[7] Agyemang C, Redekop WK, Owusu-Dabo E, Bruijnzeels M (2005). a. Blood pressure patterns in rural, semi-urban and urban children in the Ashanti region of Ghana, West Africa. BMC Public Health.

[8] Salman Z, Kirk GD, DeBoer MD (2011). High rate of obesity-associated hypertension among primary schoolchildren in Sudan. Int J Hypertens.

[9] Reilly JJ, Kelly J (2011). Long-term impact of overweight and obesity in childhood and adolescence on morbidity and premature mortality in adulthood: systematic review. Int J Obes.

[10] Singh AS, Mulder C, Twisk JWR, Van Mechelen W, Chinapaw MJM (2008). Tracking of childhood overweight into adulthood: a systematic review of the literature. Obes Rev.

[11] Swinburn B, Egger G, Ph D, Raza F. Dissecting Obesogenic Environments : The Development and Application of a Framework for Identifying and Prioritizing Environmental Interventions for Obesity 1. Prev Med. 1999;29(570):563–70. 10.1006/pmed.1999.0585 10600438

[12] Popkin BM, Gordon-Larsen P (2004). The nutrition transition: worldwide obesity dynamics and their determinants. Int J Obes.

[13] Larouche R, Saunders TJ, John Faulkner GE, Colley R, Tremblay M (2014). Associations between active school transport and physical activity, body composition, and cardiovascular fitness: a systematic review of 68 studies. J Phys Act Health.

[14] Li L, Zhang S, Huang Y, Chen K (2017). Sleep duration and obesity in children: a systematic review and meta-analysis of prospective cohort studies. J Paediatr Child Health.

[15] Chen X, Beydoun MA, Wang Y (2008). Is Sleep Duration Associated With Childhood Obesity ? A Systematic Review and Meta-analysis. Obesity (Silver Spring).

[16] Katzmarzyk PT, Barreira TV, Broyles ST, Champagne CM, Chaput JP, Fogelholm M (2015). Relationship between lifestyle behaviors and obesity in children ages 9–11: Results from a 12-country study. Obes (Silver Spring).

[17] Huang J-Y, Qi S-J (2015). Childhood obesity and food intake. World J Pediatr.

[18] Candib LM (2007). Obesity and diabetes in vulnerable populations: reflection on proximal and distal causes. Ann Fam Med.

[19] Newby PK (2007). Are dietary intakes and eating behaviors related to childhood obesity? A comprehensive review of the evidence. J Law Med Ethics.

[20] Ogden CL, Lamb MM, Carroll MD, Flegal KM (2010). Obesity and socioeconomic status in children and adolescents: United States, 2005-2008. NCHS Data Brief.

[21] Ziraba AK, Fotso JC, Ochako R (2009). Overweight and obesity in urban Africa: a problem of the rich or the poor?. BMC Public Health.

[22] Fruhstorfer BH, Mousoulis C, Uthman OA, Robertson W (2016). Socio-economic status and overweight or obesity among school-age children in sub-Saharan Africa - a systematic review. Clin Obes.

[23] Ghana Statistical Service. Ghana Demographic and Health Survey Preliminary Report 2008. 2009;

[24] Aryeetey R, Lartey A, Marquis GS, Nti H, Colecraft E, Brown P (2017). Prevalence and predictors of overweight and obesity among school-aged children in urban Ghana. BMC Obes.

[25] Mogre V, Gaa PK, Nagumsi R, Abukari S (2013). Overweight , Obesity and Thinness and Associated Factors Among School- Aged Children ( 5–14 Years ) in Tamale. Eur Sci J.

[26] Ochola S, PK iny M (2014). Dietary intake of schoolchildren and adolescents in developing countries. Ann Nutr Metab.

[27] Peltzer K, Pengpid S (2011). Overweight and obesity and associated factors among school-aged adolescents in Ghana and Uganda. Int J Env Res Public Heal.

[28] Pengpid S, Peltzer K (2015). Overweight and Obesity and Associated Factors among School-Aged Adolescents in Six Pacific Island Countries in Oceania. Int J Env Res Public Heal.

[29] Mohammed H, Vuvor F (2012). Prevalence of childhood overweight/obesity in basic school in Accra. Ghana Med J.

[30] Ghana Statistical Service. Adentan Municipality: Population and housing census. Vol. 2, District Analytical Report. 2014.

[31] Ghana School Survey (2012). Nutrition and obesity report of the Ghana school survey results dissemination workshop, September 2012.

[32] Daniel WW (1999). Biostatics; a foundation for analysis in the health sciences.

[33] Onge ES, Miller SA, Motycka C, DeBerry A (2015). A review of the treatment of type 2 diabetes in children. J Pediatr Pharmacol Ther.

[34] Kowalski KC, Crocker PRE, Donen RM. The Physical Activity Questionnaire for Older Children (PAQ-C) and Adolescents (PAQ-A) Manual. Coll Kinesiol Univ Saskatchewan. 2004:1–37.

[35] Voss C, Ogunleye AA, Sandercock GRH (2013). Physical activity questionnaire for children and adolescents: English norms and cut-off points. Pediatr Int.

[36] Hirshkowitz M, Whiton K, Albert SM, Alessi C, Bruni O, DonCarlos L (2015). National sleep foundation’s sleep time duration recommendations: methodology and results summary. Sleep Heal.

[37] De Onis M, Onyango AW, Borghi E, Siyam A, Nishida C, Siekmann J (2007). Development of a WHO growth reference for school-aged children and adolescents. Bull World Heal Organ.

[38] Filmer D, Pritchett L (2001). Estimating wealth effects without expenditure data--or tears: an application to educational enrollments in states of India. Demography..

[39] Mwaikambo SA, Leyna GH, Killewo J, Simba A, Puoane T (2015). Why are primary school children overweight and obese? A cross sectional study undertaken in Kinondoni district, Dar-es-salaam. BMC Public Health.

[40] McKersie J, Baard ML (2014). Obesity in 7–10-year-old children in urban primary schools in Port Elizabeth. South African J Sport Med.

[41] Muthuri SK, Wachira LJ, Onywera VO, Tremblay MS (2014). Correlates of objectively measured overweight/obesity and physical activity in Kenyan school children: results from ISCOLE-Kenya. BMC Public Health.

[42] Sanigorski AM, Bell C, Kremer PJ, Swinburn B (2007). High childhood obesity population in an Australian population. Obesity.

[43] Ajayi EO, Elechi HA, Alhaji MA. Prevalence of overweight / obesity among primary school pupils in Urban Centre. Nigeria. 2015:59–65.

[44] Hanson MD, Chen E (2007). Socioeconomic status and health behaviors in adolescence: a review of the literature. J Behav Med.

[45] Dinsa GD, Goryakin Y, Fumagalli E, Suhrcke M. Obesity and socioeconomic status in developing countries : a systematic review. Obes Rev. 2012:1067–79. 10.1111/j.1467-789X.2012.01017.x PMC3798095 22764734

[46] Rogers R, Eagle TF, Sheetz A, Woodward A, Leibowitz R, Song M (2015). The relationship between childhood obesity, low socioeconomic status, and race/ethnicity: lessons from Massachusetts. Child Obes.

[47] Gupta N, Goel K, Shah P, Misra A (2012). Childhood obesity in developing countries: epidemiology, determinants, and prevention. Endocr Rev.

[48] Drewnowski A. Obesity, diets, and social inequalities. Nutr Rev. 2009;67(SUPPL. 1):S36-S39. 10.1111/j.1753-4887.2009.00157.x 19453676

[49] Drenowatz C, Eisenmann JC, Pfeiffer KA, Welk G, Heelan K, Gentile D, et al. Influence of socio-economic status on habitual physical activity and sedentary behavior in 8- to 11-year old children. BMC Public Health. 2010;10. 10.1186/1471-2458-10-214 PMC2873582 20423487

[50] Sarmiento OL, Lemoine P, Gonzalez SA, Broyles ST, Denstel KD, Larouche R (2015). Relationships between active school transport and adiposity indicators in school-age children from low-, middle- and high-income countries. Int J Obes Suppl.

[51] Larouche R, Lloyd M, Knight E, Tremblay MS. Relationship Between Active School Transport and Body Mass Index in Grades- 2011;322–30. 10.1123/pes.23.3.322 21881153

[52] Denstel KD, Broyles ST, Larouche R, Sarmiento OL, Barreira TV, Chaput J-P (2015). Active school transport and weekday physical activity in 9–11-year-old children from 12 countries. Int J Obes Suppl.

[53] Tremblay MS, Gray CE, Akinroye K, Harrington DM, Katzmarzyk PT, Lambert EV (2014). Physical activity of children: a global matrix of grades comparing 15 countries. J Phys Act Health.

[54] Hart CN, Cairns A, Jelalian E (2012). Sleep and obesity in children and adolescents. Pediatr Clin N Am.

[55] Rosi A, Calestani MV, Parrino L, Milioli G, Palla L, Volta E (2017). Weight status is related with gender and sleep duration but not with dietary habits and physical activity in primary school Italian children. Nutrients.

[56] Morrissey B, Malakellis M, Whelan J, Millar L, Swinburn B, Allender S (2016). Sleep duration and risk of obesity among a sample of Victorian school children. BMC Public Health.

[57] Cain N, Gradisar M (2010). Electronic media use and sleep in school-aged children and adolescents: a review. Sleep Med.

[58] Westerlund L, Ray C, Roos E (2009). Associations between sleeping habits and food consumption patterns among 1011-year-old children in Finland. Br J Nutr.

[59] Weiss A, Xu F, Storfer-Isser A, Thomas A, Ievers-Landis CE, Redline S (2010). The association of sleep duration with adolescents’ fat and carbohydrate consumption. Sleep..

[60] Chaput J-P, Tremblay A (2012). Insufficient sleep as a contributor to weight gain: an update. Curr Obes Rep.

[61] Braithwaite I, Stewart AW, Hancox RJ, Beasley R, Murphy R, Mitchell EA. The Worldwide Association between Television Viewing and Obesity in Children and Adolescents: Cross Sectional Study. PLoS One. 2013;8(9). 10.1371/journal.pone.0074263 PMC3783429 24086327

[62] Peck T, Scharf RJ, Conaway MR, DeBoer MD (2015). Viewing as little as 1 hour of TV daily is associated with higher change in BMI between kindergarten and first grade. Obesity.

[63] Zhang G, Wu L, Zhou L, Lu W, Mao C (2016). Television watching and risk of childhood obesity: a meta-analysis. Eur J Pub Health.

[64] Tremblay MS, LeBlanc AG, Kho ME, Saunders TJ, Larouche R, Colley RC (2011). Systematic review of sedentary behaviour and health indicators in school-aged children and youth. Int J Behav Nutr Phys Act.

[65] Dutra GF, Kaufmann CC, Pretto ADB, Albernaz EP (2015). Television viewing habits and their influence on physical activity and childhood overweight. J Pediatr.

[66] Crespo CJ, Smit E, Troiano RP, Bartlett SJ, Macera CA, Andersen RE (2001). Television watching, energy intake, and obesity in US children. ArchPediatrAdolescMed.

[67] Lobstein T, Dibb S (2005). Evidence of a possible link between obesogenic food advertising and child PubMed commons. Obes Rev.

[68] Hu FB (2013). Resolved: there is sufficient scientific evidence that decreasing sugar-sweetened beverage consumption will reduce the prevalence of obesity and obesity-related diseases. Obes Rev.

[69] Zakrzewski JK, Gillison FB, Cumming S, Church TS, Katzmarzyk PT, Broyles ST (2015). Associations between breakfast frequency and adiposity indicators in children from 12 countries. Int J Obes Suppl.

[70] Mushtaq MU, Gull S, Mushtaq K, Shahid U, Shad MA, Akram J (2011). Dietary behaviors, physical activity and sedentary lifestyle associated with overweight and obesity, and their socio-demographic correlates, among Pakistani primary school children. Int J Behav Nutr Phys Act.

[71] Ocansey R, Aryeetey R, Sofo S, Delali MB, Pambo P, Nyawornota VK (2014). Results from Ghana’s 2014 report card on physical activity for children and youth. J Phys Act Health.

[72] Heitmann BL, Lissner L (1995). Dietary underreporting by obese individuals-is it specific or non-specific?. BMJ..

